# Improving quality and safety of cancer care for people from ethnic minority backgrounds: what do consumers want?

**DOI:** 10.1007/s00520-025-09665-6

**Published:** 2025-06-27

**Authors:** Bronwyn Newman, Ashfaq Chauhan, Thit Tieu, Margo Van Poucke, Carlene Wilson, Ramesh Lahiru Walpola, Elizabeth Manias, Holly Seale, Desiree Leone, Allan Ben Smith, Melvin Chin, Nadine Elkabbout, Reema Harrison

**Affiliations:** 1 Centre for Health Systems and Safety Research, Australian Institute of Health Innovation, Macquarie University, Macquarie Park, NSW Australia; 2 CanEngage Consumer Group, Centre for Health Systems and Safety Research, Australian Institute of Health Innovation, Macquarie University, Macquarie Park, NSW Australia; 3 Melbourne School of Population and Global Health, University of Melbourne, Carlton, VIC 3053 Australia; 4 School of Health Sciences, UNSW Sydney, Kensington, NSW Australia; 5 School of Nursing and Midwifery, Monash University, Clayton, VIC Australia; 6 School of Population Health, UNSW Sydney, Kensington, NSW Australia; 7 Multicultural Health Services, Western Sydney Local Health District, North Parramatta, New South Wales Australia; 8 The Daffodil Centre, The University of Sydney, a joint venture with Cancer Council NSW, Camperdown, NSW Australia; 9 Medical Oncology, Prince of Wales Hospital, South Eastern Sydney Local Health District, Randwick, NSW Australia

**Keywords:** Patient engagement, Ethnic minority, Cancer care, Patient participation

## Abstract

**Objectives:**

People from ethnic minority backgrounds have increased likelihood of poor care quality and outcomes than the general population when accessing cancer care. Consumer-centric solutions are required to address these inequities. We explored the views of people from ethnic minority backgrounds on key issues impacting their cancer care and outcomes, including strategies to enhance care.

**Design:**

Qualitative semi-structured interviews were conducted to collect experiential data from people from diverse backgrounds who had accessed cancer care. All recorded interviews were transcribed verbatim, and one was included with notes only. Thematic analysis was conducted, with initial coding by two researchers and themes further developed by all authors.

**Results:**

Interviews were conducted with 19 consumers, including six patients and 13 support persons who had accessed cancer care from hospitals in NSW and Victoria, Australia. Participants were from diverse cultural backgrounds including Vietnamese, Chinese, Iraqi and Filipino, and nine interviews were conducted with language support. Consumers indicated that cancer services need to develop flexible care systems to improve care, expressed through three themes: (i) considering language and culture, (ii) establishing communication and (iii) accommodating difference.

**Conclusion:**

Current practices leave people from ethnic minority backgrounds more vulnerable to compromises in quality and safety of care. Involving people from ethnic minority communities relevant to the local area in service design and evaluation is essential to inform practice change. Further consideration is required to address resourcing issues and provide governance support to enable care that is flexible and equipped to respond to varied language and cultural support needs.

**Supplementary Information:**

The online version contains supplementary material available at 10.1007/s00520-025-09665-6.

## Introduction

People from ethnic minority backgrounds accessing cancer care experience higher rates of patient safety events, including health care-acquired infections and medication errors, compared to the general population both in Australia, and internationally [1]. The elements contributing to this disparity are complex, often linked to factors such as shared language (with health professionals), health literacy, socioeconomic factors, length of time in their country of residence and social engagement [1]. People from ethnic minority backgrounds include people who were born overseas or have one or more parents born overseas, speak a non-mainstream language, practice a religion, culture or hold a faith outside the national mainstream religion, culture or faiths [2]. Understanding the perspectives of consumers from ethnic minority backgrounds is essential to identifying features of health services and systems that can fail to meet the needs of ethnic minority populations receiving treatment for cancer and opportunities for improvement [3].

Australia has a diverse population. The 2021 census found 27.6% of the Australian population were born overseas and more than half (51.5%) of the population had at least one parent born overseas, with 22% of Australians using a language other than English at home [4]. Patients receiving cancer care are at increased risk of harm when compared to general hospital patients, during or after their acute hospital care, and patients from ethnic minority backgrounds are known to be exposed to patient safety events more frequently [5]. A retrospective review of 628 medical records of patients from ethnic minority backgrounds conducted across four cancer services in Australia noted that one in three of these patients was exposed to a patient safety event [2].

Many factors come together to create vulnerability for people from ethnic minority backgrounds when accessing health care, and these are often linked to broader systemic barriers that impact effective communication and care [6–8] Health services are often embedded within large systems ill-equipped to meet the needs of people from diverse backgrounds, with administrative and care processes designed to suit the majority or ‘norm’ [9]. Barriers to health care access that impact people from ethnic minority backgrounds [6, 9], and more specifically cancer care [10–12], have been described in recent studies. Traditionally, vulnerability and access challenges have been attributed to individual or personal characteristics, whereas, more recently, the role and responsibility of services to provide suitable care to diverse groups have been recognised [13]. The responsibility of health service agencies to accommodate diverse cultural needs of consumers has been acknowledged in Australia and internationally [8] with recent calls for effective strategy development advocating for codesign.

Despite increased awareness of the need for change, there is little in the peer-reviewed literature that describes an exploration of ethnic minority consumers’ views about the elements of health care provision in the cancer services setting that impact quality and safety of care [14, 15]. The term ‘consumer’ incorporates patients, advocates or supporters, including family members [16, 17]. Patient survey data in Australia and internationally highlights issues related to poor communication between providers and patients [18] and reports lower overall satisfaction, and less positive health care experiences, amongst ethnic minority people than the general population accessing health care [15, 19]. The limited research on the needs and perspectives of ethnic minority consumers accessing cancer care is not indicative of the level of advocacy for change, or recommendations for adaptation of care [10, 20, 21]. In this research, we sought consumer perspectives to add to the current knowledge base. To this end, we explored the views of ethnic minority consumers on the key issues that impacted their experience of cancer care, and attempted to identify strategies that could enhance care and improve safety, guided by two key questions:

- What are the key issues that services need to consider to improve safety in cancer care for people from ethnic minority backgrounds?
- What can health services do to improve care quality and outcomes for people from ethnic minority backgrounds?

## Methods

Ethics approval was granted by a National Health and Medical Research Council accredited Human Research Ethics Committee (Reference—2020/ETH00965) in accordance with the Declaration of Helsinki. This study is reported in accordance with the Consolidated Criteria for Reporting Qualitative Studies guidelines [22].

### Design

A cross-sectional descriptive qualitative study was undertaken using semi-structured interviews to collect experiential data from people from ethnic minority backgrounds who had accessed cancer care as patients or their supporters, including family members.

### Setting

This study was embedded in a larger project conducted across four cancer services in the state capitals of two Australian states: New South Wales (two services) and Victoria (two services) [22]. All four cancer services were based within publicly funded, teaching hospitals that ranged in size from 450 to 670 beds. All provided chemotherapy, radiotherapy, immunotherapy and palliative care services across inpatient and outpatient cancer care settings.

### Recruitment

Participants were eligible if they had accessed cancer care as a patient or had supported a patient at the participating cancer services in the preceding 5 years, were over 18 years and self-identified as coming from an ethnic minority background. Recruitment was conducted within participating cancer services using purposive sampling and via consumer organisation networks, social media and a specialist ethnic minority recruitment agency. Interpreters or bilingual field workers were available, as required. Consent forms were available in translated formats. Written consent to participate was obtained prior to the interview with verbal consent also confirmed at interview commencement.

### Interview process

Semi-structured interviews were conducted in person, online or via telephone by three researchers unknown to participants (KJ;AC; BN). Data collection was conducted from September 2021 to June 2023 and interviews lasted between 28 and 77 min (average 38 min) and one participant chose not to have the interview recorded and analysis included researcher notes from this interview. We opted to use semi-structured interviews scaffolded by an interview guide because this method offers a flexible approach to tailor interactions and explore issues as they arise [23]. The interview guide was developed collaboratively with members of the research team experienced in conducting qualitative research and members of the project consumer advisory group informed by previous research and experience (Supplementary file 1).

### Data analysis

Audio recordings of interviews conducted in English were transcribed verbatim by an experienced research assistant (MvP), and recordings of interviews with interpreter support were transcribed by a professional transcription service. To maintain anonymity, participant codes were generated with site, gender and ‘carer or patient’ indicated in coding label. ‘Carer’ indicated a person in an unpaid caring role, often a family member. Data were inductively analysed using thematic analysis with reference to the social ecological model [24]. Coding was an iterative process, and the two researchers met weekly to discuss findings until coding was complete. A subset of transcripts was reviewed by other team members (EM, DL, CW, HS, AC and RH) and the findings discussed as a group. Following this, themes were identified, with preliminary themes defined and refined in consultation with consumer authors (NE, TT). Final themes were checked by all research team members to ensure rigour.

### Findings

A total of 19 consumer interviews were conducted across Victoria and NSW. Participant characteristics are presented in Table 1.

**Table 1:** Participant characteristics

Location	Gender	Ethnicity/language group identified	Language support	Patient or family member	Interview length (min)
NSW	F	Vietnamese	No	Patient	30
NSW	F	Arabic speaking	No	Patient	41
NSW	F	Arabic speaking	Yes—interpreter	Patient	29
NSW	F	Arabic Speaking	Yes—interpreter	Patient	37
NSW	F	Arabic speaking, Iraq	Yes—interpreter	Patient	48
NSW	M	Arabic speaking, Iraq	Yes—bilingual field worker	Patient	39
NSW	M	Arabic speaking	Yes—interpreter	Family member	41
NSW	F	Ukrainian	No	Family member	46
NSW	M	Chinese, Mandarin	Yes—bilingual worker	Patient	76
NSW	M	Chinese, Mandarin	Yes—bilingual worker	Patient	65
NSW	F	Arabic	No	Family member	NOTES ONLY—no recording
Vic	F	India	No	Patient	24
Vic	F	Arabic speaking, Iraq	Yes	Patient	33
Vic	M	Iraq	No	Family member	31
Vic	F	Filipino	No	Family member	42
Vic	F	China	No	Patient	28
Vic	F	China	Yes—interpreter	Patient	38
Vic	F	China	No	Family member	35
Vic	F	China	Yes—interpreter	Patient	32

Participants reported that consumers from ethnic minority backgrounds are often influenced by experiences that compound the stress of a cancer diagnosis, and the rigours of cancer treatment. Three themes were developed from the data with 11 subthemes (Supplementary File 2).

#### Theme 1: Considering language and culture in cancer care

**Fig. 1:**
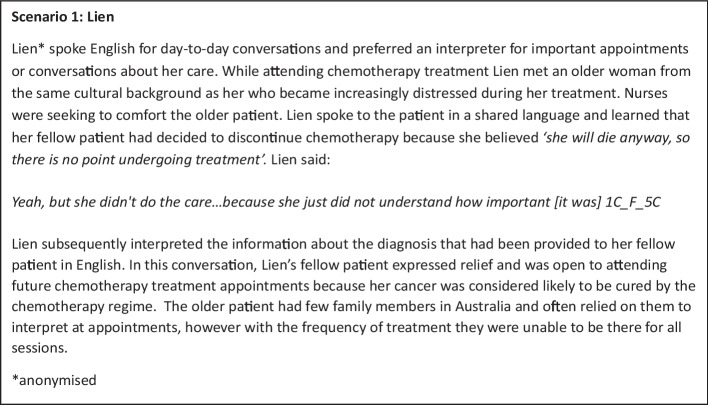
Lien’s experience

Language and culture were often discussed simultaneously by participants and viewed as interrelated, impacting opportunities to engage with practitioners, appraise treatment options and plan care. We commence the exploration of theme 1 with a brief description of an experience described by one of our interview participants in Fig. 1.

The experience described in Fig. 1 illustrates the interplay between the four subthemes: the impact of culture on communication about a cancer diagnosis, value of culturally congruent support, impact of immigration on available supports and complexity of asking questions or raising issues. These four subthemes were explored in relation to all interview data and relevant quotes are presented in relation to each subtheme in Table 2 and indicated by participant number in the text.

**Table 2:** Theme 1: Considering language and culture in cancer care

Theme 1: Considering language and culture in cancer care		
*Subtheme*	*Participant ID*	*Quote*
*Interpretation of a cancer diagnosis*	*(7C_M_5C)*	*in our culture, our countries where someone has cancer it’s just we understand that this is a terminal illness and that made—just the word cancer makes things really, really hard for all of us*
	*(2 F 5C*)	*I think they [health services] need to know first of all, because the name cancer in a lot of our communities and everywhere, I guess, means death*
*Support from people with a shared cultural background is valuable*	(8C_F_1A)	*…she has a lot of anxiety. I think anxiety more from the point that coming from a different background, not having—not being able to speak English, converse with the doctors…*
	(15C_F_3C)	*I mean, if Asian doctor is there, I’m really happy. I can ask more questions, because we have the same background….For discuss with my GP at the beginning is very good, because she speak Mandarin same like me. So I can ask a lot of questions,*
	(1C_F_5C)	*…the first appointment or second are important. So I’d really need my daughter with me so so they can, she can now to know what exactly they would do to, and even emotional support as well*
	(2C F 5 C)	*I took two of my daughters and my husband with me. Ah, because at that stage I don’t think I could take all the information the same. But I think a support person. They could ask questions that I wouldn’t think about*
	(1C_F_5C)	*Luckily I got my daughter and my son and my son in law at home. They did help me out through the emails to go through everything*
*Isolation can be a feature of immigration that impacts access to support*	(9C_M_1A)	*I don’t have any family member near with me. Also, all the family member is overseas. Not in Australia*
	(8C_F_1A)	*One of the reasons they never took the ability to speak English was because they never had anyone to communicate with*
*Raising issues or asking questions can be challenging*	(16C_F_3C)	*I think she tried to ask questions herself 2 or 3 times, but it’s just they couldn’t understand, so it decreased her willingness to communicate*
	(9C_M_1A)	*Sometimes I feel shame. I feel embarrassed to ask Him [Doctor] ….Actually, I do but I feel shame to ask anymore………I do not feel free to call the doctor or call the nurse through the phone number which is the hospital give to me*
	(10_M_1A)	*He (grandfather) usually doesn’t have questions because he won’t ask anything. He will just listen to the doctor says*

##### **Subtheme 1.1: Interpretation of a cancer diagnosis**

Most participants spoke about the feeling of ‘difference’ as they navigated a cultural understanding of cancer that was not always shared or understood by cancer care staff (illustrated in Fig. 1). Our data reflected that for many participants, cancer was seen as a ‘shame’ or ‘death sentence’, and a poor outcome was considered a certainty (7C_M_5C). Participants reported that services were not always equipped to deal with the cultural nuance in interpretation of a cancer diagnosis and this was often exacerbated by lack of shared language (2 F 5 C).

##### **Subtheme 1.2: Support from people with a shared cultural background is valuable**

Support from people with shared cultural background was identified as particularly important for both language and emotional support as seen in Lien’s experience. Participants all reported experiences of anxiety and shock at the overwhelming nature of their diagnosis, which they saw as somewhat expected. For many, this experience was accompanied by the compounding stress of limited communication options (8C_F_1A_Carer). Participants highlighted their comfort in receiving care from a practitioner with a shared culture or language, and five participants relayed positive experiences with their GP (15C_F_3C). The role of family support in cancer diagnosis, throughout treatment and ongoing care, was recognised as important across various cultures represented (1C_F_5C), (2C F 5 C), (1C_F_5C). The impact of an absence of family support was also identified; this impact was magnified when family had immigrated recently or had limited community connection.

##### **Subtheme 1.3: Isolation can be a feature of immigration that impacts access to support**

Some participants identified isolation as a key feature of migration, particularly for older people accessing cancer care. Consumers expressed distress, describing a lack of shared community for support during cancer diagnosis and treatment (9C_M_1A). Isolation and limited social connection impacted the ability of consumers to access care and understand health systems and health information (8C_F_1A).

##### **Subtheme 1.4: Raising issues or asking questions can be challenging**

**Fig. 2:**
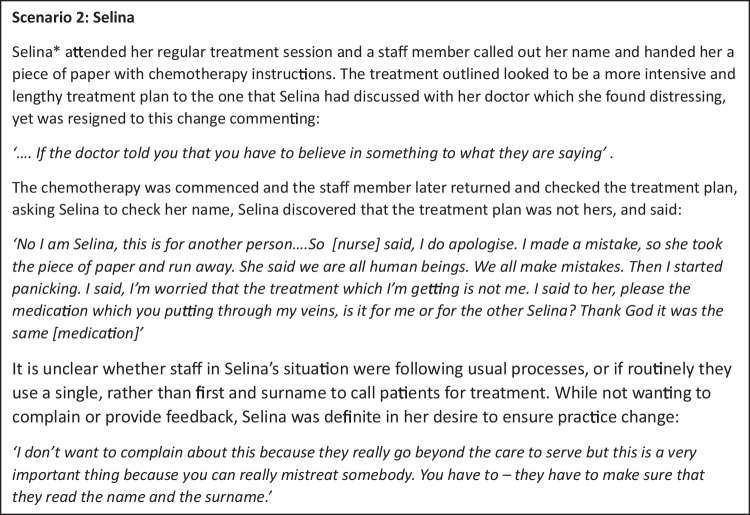
Selina’s experience

Lien’s experience also illustrates the impact of limited access to language support during consultations with oncology staff. Participants identified barriers to interaction between consumers and health care staff, suggesting that initial failure to communicate impacted ongoing confidence in interactions and confidence in asking questions (16C_F_3C). The hesitancy to questions and belief in the doctor’s authority were commonly reported barriers to communication (9C_M_1A), (10_M_1A); Selina’s experience (Fig. 2) further illustrates this theme.

#### Theme 2: Facilitating opportunities to establish communication

Building on theme 1, participants identified that communication was of central importance when accessing cancer care. Although some participants highlighted positive communication experiences, many identified situations where communication was compromised. This theme emphasises the importance of clarity in negotiating information access and the practicalities of booking and using interpreters. Supporting quotes are included in Table 3.

**Table 3:** Theme 2: Facilitating opportunities to establish communication

Theme 2: Facilitating opportunities to establish communication		
*Subtheme*	*Participant ID*	*Quote*
*Negotiating appropriate communication options*	(15C_F_3C)	*She [doctor] just finds a related photos and explain to me. Okay, that’s really helpful*
	(14C_F_4D)	*I think you’re the one that’s, if you’re the one that’s dying, I think it’s only fair that you’re the one that knows first or gets told the news first before the people that get affected*
	(16C_F_3C)	*Well, they assume that I will interpret things for her*
	(15C_F_3C)	*He [oncologist] asked me if you are not confident that doesn’t matter just to use interpreter. But he also said, okay, I think you are okay. We can communicate*
	(15C_F_3C)	*If at that part, if interpreter can get more involved, I can know the more information*
*Enabling use of interpreters*	*(3F_F_5C)*	*Because the doctor tell me you have to—they give you an interpreter because they’ve got something you don’t understand about…*
	*(9C_M_1A)*	*So when my doctor take the meeting or seminar or discuss in front of me for my illness, like the oncologist is a chemotherapy doctor and radiation therapy doctor, they discuss all my details of my illness in front of the computer. Explain. I was in front of them. I don’t understand what do you think because there’s too much terminology*
	(4_F_5C)	*In all the stages mostly I did need an interpreter, but I was always worried that the interpreter will stop or getting an interpreter quickly might hinder the urgency of the service….I was worried that my treatment would be late because of it, getting an interpreter*
	(1C_F_5C)	*They ask me [if I need an interpreter] and I say I’m OK cause I don’t want to wait and it’s usually really late/delayed appointment*
	(15_C_F_3C)	*I try to book an interpreter. But if you was a lot of time… So I just skip that step after I just book… I just use my dictionary*
*Translated information to support understanding and informed choice*	(9C_M_1A_)	*So this is a lot of information that consider maybe to give the think—the thought, to give them plenty or enough, very comprehensive information to the patient but, for the people from the non-English background, non-Australia background, the speaker actually is not enough. Far not enough. This is not effective*
	(15_C_F_3C)	*I want to know the more information, no matter, good or not good… What, what should I do next?*
	*(9C_M_1A_)*	*I have the right. I’m supposed to be entitled to understand what are we going to do before you make a decision to*
	*(16C_F_3C)*	*I think if information translated that’s available, maybe on the website, or maybe in like a QR. Code kind of thing it will be. It will be much more helpful*
	*(9C_M_1A)*	*I feel a little scared but no one tell me, what is the plasma exchange? I double check from my dictionary. My dictionary is not a medical dictionary, I cannot find out what it exactly means in Chinese*
*Health service contact points between appointments*	(11C_F_4D)	*Whenever I’m sending any emails to my nurse she is replying me very quickly…..you get the response very quickly ….I will get a reply straight away. It was very easy to communicate*
	(8C_F_1A)	*So you need someone who can see you fairly quickly so that it’s not just the smooth sailing. It’s not a smooth road. You need to be able, if anything like this happens, you need to have the number …. It’s just communicating in between the appointments if there were side issues like her—especially with this stomach, it became a huge issue for her…*
	(10_M_1A)	*When he [patient] started showing these severe side effects, the oncologist I think is on leave, and [PATIENT] thinks it’s not polite to call someone when they are on leave, you know. So he didn’t*

##### **Subtheme 2.1: Negotiating appropriate communication options**

Participants’ diverse cultural and socio-economic backgrounds (see Table 1) were associated with differences in the availability of support. The clear message from participants was that it was essential to establish a personally appropriate conduit for information and communication to ensure messaging was accurate and timely. Participants consistently suggested that providing access to a range of options to support communication, during and between appointments, was key to improving current services. Some participants appreciated use of visual aids or pictures (15C_F_3C). Many participants valued using family members as interpreters but also indicated that this is not appropriate in all circumstances. One family member discussed the discomfort of providing language support conveying difficult news to her grandfather about his diagnosis. Relaying the importance of using professional interpreters in making sure that the patient receives the news first (14C_F_4D). Similarly, another family member spoke about the pressure of providing language support reporting that service providers assumed this was her role (16C_F_3C).

Participants expressed a desire for systems equipped to accommodate variation in their requirements for interpreters across different tasks or appointment types. Some people indicated that they preferred an interpreter only for key appointments such as diagnosis. Others valued the opportunity to negotiate the presence of an interpreter (15C_F_3C). Much of the commentary about interpreter use related to systemic barriers to availability and expressing that health system improvement is required to enable access to interpreters as needed.

##### **Subtheme 2.2: Enabling use of interpreters**

Comments about interpreter availability, systems for booking an interpreter and the impact of difficulties in interpreter access were reported by participants from all four sites. Access to interpreters was often dependent on the health care provider (3F_F_5C)*.*

Access to interpreters for initial appointments or planned specialist visits was widely reported; however, outside of these activities, participants were less clear about how, when or whether to access support. For example, one participant commented that during medical ‘rounds’, the option of an interpreter was not provided (9C_M_1A).

The impact of limited availability and booking complexities resulted in apprehension about accessing language support. Participants commented that they feared asking for an interpreter may delay treatment and therefore chose not to access support (4_F_5C), (1C_F_5C) (15_C_F_3C).

##### **Subtheme 2.3: Translated information to support understanding and informed choice**

Four consumers expressed a desire for translated, evidence-based information, particularly at the time of diagnosis and treatment planning (9C_M_1A_). Translated information in varied formats (16C_F_3C) was thought important because it helped patients understand their diagnosis, consider their treatment options and discuss these with the family after the appointment (15_C_F_3C). Participants indicated that they did not always receive sufficient information to support an informed treatment choice, and recognised that they have a right to access the appropriate information (9C_M_1A_).

Another consumer gave a specific example of limited access to translated information when they described being asked to decide about undergoing a plasma exchange (9C_M_1A). *Limited* access to information impacted wellbeing, increased anxiety and decreased confidence to ask questions.

##### **Subtheme 2.4: Health service contact points between appointments**

A small number of people highlighted the benefit of clear systems for contact between appointments (11C_F_4D). Participants spoke about the benefits of immediate responses to important questions (8C_F_1A) and other participants found the lack of clear health service pathways for care between appointments difficult, with this identified as a source of stress. One family member identified the patient’s hesitancy to phone the oncologist, illuminating the need for clear instruction about who to contact in various circumstances (10_M_1A).

#### Theme 3: Improving the ability of health systems to accommodate difference

In addition to the challenges and considerations identified in themes 1 and 2, participants frequently shared examples of care that was complicated or compromised by health service processes or requirements. Theme 3 explores these health service processes or systems, identifying barriers to effective cancer care for ethnic minority consumers, along with potential strategies for improvement. Participants reported that health care systems were often not underpinned by policy designed or equipped to accommodate diverse cultural and language needs. Recognising the risks created by limited access to information, supports and services, one consumer talked about the urgency of this need to be communicated to policy makers (9C_M_1A_).

Three subthemes were identified related to intractability of the administrative processes, information about available supports and integrating culturally appropriate support options and supporting quotes are presented in Table 4.

**Table 4:** Theme 3: Improving the ability of health systems to accommodate difference

Theme 3: Improving the ability of health systems to accommodate difference		
*Subtheme*	*Participant ID*	*Quote*
	*(9C_M_1A_)*	*(We need) …feedback to the government immediately to help the policy maker to adjust their policy or decision. Immediately. Instead of they change the decision until the accident happens*
*Intractability of administrative processes*	(1C_F_5C)	*When I went to [HOSPITAL], the waiting times so long, so overcrowded. One time me and my daughter waiting from morning ‘til afternoon – whole day*
*Information about services*	*(2C_F_5C)*	*What I’m saying, because her English wasn’t that good [and there were no simple options], and her daughter and son, they were the one who’s taking charge of asking questions, and you know, doing all that need somebody to explain. (2C F 5 C)*
	*(2C_F_5C)*	*We need people on the team if you, if you people like uh a dietician. Ah! Because what I did for myself then I just went on looking for video clips everywhere. People who had cancer all over the world. What did they eat? What did they do? What was good? Because no one told me*
*Culturally appropriate supports*	*(2C_F_5C)*	*…psychology, but I think with our culture it’s not appropriate. We don’t really feel engaged with the um psychologist* *I was thinking in there like once somebody is diagnosed, and they go and see someone like afterward to talk to someone who has maybe someone who has been through the yeah, through it, or somebody that there is a support there that somebody can talk to them with the same language, and you know, look at the fear they have and work with them and with the team, too. ….. If there is somebody who speak the language? Not a ah, not an interpreter, because they can only say what they hear and interpret. They’re not allowed to converse*
	(1C_F_5C)	*I’m Vietnamese. I want to talk to the Vietnamese person, the same age as me, with me the same situation with me, you know, like we can talk like, you know…..This way it allows the carer or the patient to ask any question that arise after each session*

##### **Subtheme 3.1: Intractability of administrative processes**

Two examples of the intractability of administrative processes for non-English speakers were highlighted. The first was around appointment schedules and bookings with six participants describing appointment booking systems that were difficult to navigate, particularly when seeking to include family. The critical need for family support when interacting with the health system was noted, and the impact of long waiting times was a particular concern (1C_F_5C).

The second example of administrative difficulties that arose in our data was around name checking and medication. Participants reported that community members from their cultural group often share similar names that are unfamiliar to health care workers and this resulted in confusion. Selina’s experience, outlined in the text box below, illustrates the potential implications.

As in Selina’s situation (Fig. 2), none of the participants in our study, who received wrong medication or ‘near misses’, raised this issue with staff and did not intend to do so. All expressed gratitude for the care received and several participants from various cultural backgrounds identified hesitancy about raising concerns. Nonetheless, they all noted the need for a clearer pathway to share feedback, if they wished to do so. This theme connects with subtheme 1.4, reinforcing the need for clarity in systems to raise issues or concerns.

##### **Subtheme 3.2: Information about services**

Many participants described being unfamiliar with the health system and said they were offered limited information about supports available outside the medical care they were accessing. We found that services were often available, yet the pathway to care was obscure to those who required access. This included other treatment options and community, or allied health supports. One participant was strong in her advocacy for provision of simple, evidence-based information for people to make decisions about care, whether English or non-English speakers, highlighting the experience of a friend in similar circumstances (2C_F_5C). This participant also raised the issue of limited information about access to allied health or support services.

##### **Subtheme 3.3: Culturally appropriate supports**

Participants not only highlighted the need for information about emotional and allied health supports but also emphasised the need for culturally specific supports such as bilingual counselling services and culturally congruent peer support (1C_F_5C), (2C_F_5C). Five participants had attended a cancer education and support group conducted by bilingual staff. These consumers consistently highlighted the value of both the education and peer support that the group provided.

## Discussion

The overarching message from our interview data is that that health systems need to be designed to expect differences in language and culture within their patient cohort. The experiences described by interview participants provide rich examples of the complex array of factors that come together in health care interactions, and more specifically, cancer care. Experiences such as those illustrated in Lien and Selina’s stories illustrate how vulnerabilities created by services not attuned to cultural differences can impact care quality and safety. Consumers clearly conveyed that culture and language need to be considered in all aspects of care. Participants indicated change was needed in areas such as administrative processes (for example, name checking as in Selina’s experience), flexible appointment options to include family when preferred and interpreter booking systems that enable access as needed.

Multiple factors intersect to influence both the quality and safety of care for consumers from ethnic minority backgrounds, and this was illustrated by experiences described in this study. Numerous factors intersect for ethnic minority patients accessing cancer care, particularly factors impacting effective communication, and these have been identified in the literature [1, 8, 25]. The data from our research provides lived examples of these inter-related factors in consumer experience, enriching and strengthening the current evidence base. Additionally, participants who experienced poor outcomes, or limited access to care, all commented that they did not raise their concerns or issues with health service staff; this too is reflective of previous studies [26, 27]. The lack of reporting raises questions about the accessibility of systems in place to facilitate feedback from vulnerable patients from ethnic minority backgrounds [10, 28] and further reinforces previous studies that have identified reluctance to raise issues or provide feedback about issues of concern [29].

The importance of health services resourcing staff capacity for flexible provision of care was recognised by participants in our study across various cultural backgrounds, language groups and locations. The findings in this paper with a consumer focus complement and strengthen our previous work, particularly in relation to interpreter use [30]. The need for greater consideration of culture in cancer care [25], and systemic organisational change to reduce structural barriers to care and outcomes [6, 31, 32] is well-recognised. Our study draws attention to some key practical improvements to health care processes that could result in more equitable access care. These are as follows:

i. culturally appropriate, translated diagnosis and treatment information, including information about allied health services,
ii. clearly defined contact points to raise concerns and provide feedback, and
iii. flexible systems to support access to interpreter services, and carer support for family members or other supporters when required.

### Strengths and limitations

The participation of consumers across multiple locations was strength of this study because it enabled us to identify themes evident across diverse sites. A limitation of this approach was that we were unable to explore site-specific issues or solutions.

We acknowledge the difficulty in gaining the perspective of people who are not actively engaged with health care services. We adopted a broad recruitment approach which went some way to addressing this challenge.

The interviews in this study were conducted in person, via telephone and online via Zoom. We recognise that there are limitations in this approach because meeting in person, is more conducive to developing relationship with interview participants [33]. However, the use of Zoom and telephone was also strength as this enabled involvement with people from diverse locations or hesitancy about face to face contact due to infection risk.

## Conclusion

Cancer services require greater flexibility to tailor approaches to meet both language and cultural needs of consumers from ethnic minority backgrounds. Our data reinforced current research findings and advocacy, bolstering evidence for more culturally informed cancer care systems, particularly reform in the administration and provision of interpreter services. Further consideration of how staff can be resourced to provide flexible, responsive practice to accommodate varied language and cultural support needs is required. Involving people from relevant ethnic minority communities in service design and evaluation is essential to inform change but must also be supported by the necessary resources to implement and sustain desired changes. Policy is required to support practice that enables greater opportunity for consumers to choose how and when they engage with cultural and language support services, and more flexibility for staff to engage supports and plan care.

## Supplementary Information

Below is the link to the electronic supplementary material.

Supplementary file1 (DOCX 32 KB)

Supplementary file2 (DOCX 17 KB)

Supplementary file3 (DOCX 16 KB)

## Data Availability

No datasets were generated or analysed during the current study.
