# Correction to: Improving quality and safety of cancer care for people from ethnic minority backgrounds: what do consumers want?

**DOI:** 10.1007/s00520-025-09836-5

**Published:** 2025-08-06

**Authors:** Bronwyn Newman, Ashfaq Chauhan, Thit Tieu, Margo Van Poucke, Carlene Wilson, Ramesh Lahiru Walpola, Elizabeth Manias, Holly Seale, Desiree Leone, Allan Ben Smith, Melvin Chin, Nadine Elkabbout, Reema Harrison

**Affiliations:** 1 Centre for Health Systems and Safety Research, Australian Institute of Health Innovation, Macquarie University, Macquarie Park, NSW Australia; 2 CanEngage Consumer Group, Centre for Health Systems and Safety Research, Australian Institute of Health Innovation, Macquarie University, Macquarie Park, NSW Australia; 3 Melbourne School of Population and Global Health, University of Melbourne, CARLTON, VIC 3053 Australia; 4 School of Health Sciences, UNSW Sydney, Kensington, NSW Australia; 5 School of Nursing and Midwifery, Monash University, Clayton, VIC Australia; 6 School of Population Health, UNSW Sydney, Kensington, NSW Australia; 7 Multicultural Health Services, Western Sydney Local Health District, North Parramatta, New South Wales Australia; 8 The Dafodil Centre, The University of Sydney, a joint venture with Cancer Council NSW, Camperdown, NSW Australia; 9 Medical Oncology, Prince of Wales Hospital, South Eastern Sydney Local Health District, Randwick, NSW Australia

**Correction to: Supportive Care in Cancer (2025) 33:635**

10.1007/s00520-025-09665-6

The correct name is Allan Ben Smith, not Allen Ben Smith.

The affiliation of Carlene Wilson has been corrected, and her email has been added.

3 Melbourne School of Population and Global Health, University of Melbourne, CARLTON, VIC 3053

Email: carlene.wilson1@unimelb.edu.au

And not

3 Olivia Newton-John Cancer Research Centre, Austin Hospital, Heidelberg, VIC, Australia

4 School of Psychology & Public Health, La Trobe University, Bundoora, VIC, Australia

The affiliations have been updated accordingly.

The original article is corrected.

