# Supplementary material for: Improving quality and safety of cancer care for people from ethnic minority backgrounds: what do consumers want?
Source: Support Care Cancer. 2025 Jun 27;33(7):635. doi: 10.1007/s00520-025-09665-6 (PMC12204909; doi:10.1007/s00520-025-09665-6)
Supplement: Supplementary file 1 — Supplementary file1 (DOCX 32 KB) [file 520_2025_9665_MOESM1_ESM.docx]

**Study title: Exploring patient engagement in cancer settings for culturally and linguistically diverse (CALD) cancer consumers.**

Facilitator to:

- Describe the research, identify the research team and thank the participant for attending.
- Check that it is OK to audio-record the session
- Go through study information and address any questions
- Obtain verbal consent before starting the interview

Each topic areas will be introduced with an opening question and then follow up probes will be used to steer the interview

Interview topic guide for CALD Consumers

1. Can you tell me a bit about yourself, your background and how long you have been accessing XXX service?
2. Can you tell me a little more about your experience with the service?
   1. Has it been easy to access information about what is happening, what you need to do and to ask questions that you have had along the way? Can you provide some examples of your experience with accessing information?
   2. What has been your experience of the staff working in the service including reception and administrative staff? Can you provide some examples of your experiences in working with staff in the service?
   3. To what extent have you felt that your views and preferences have been explored and used in planning your care? Can you provide some examples of where your views and preferences have been taken into account?
   4. To what extent do you feel that having your background has shaped your care experience – and in what ways/why?
   5. Do you feel that you can speak up / raise concerns that you may have about your care? – have you ever had to do this?
3. Can you give me an example of a particular experience that stands out for you as a good experience with the service?
   1. What was so good about it?
   2. How did it impact you or your care?
   3. What did that experience suggest to you about the way that the service works with patients and families?
4. Now can you tell me about any experience or experiences that stand out as not so good?
   1. How did you deal with that experience?
   2. What were the impacts for your care?
   3. What did that experience suggest to you about the way that the service works with patients and families?
5. Based on your experience/perspective do you think there are practices in place that encourage you to talk about your care openly and easily with the health professionals and other staff in the service?
   1. What makes it easier or more difficult to talk about your care?
6. From your perspective, what changes in the service would make it easier for you or your family/carers to interact with the healthcare staff?
7. From your perspective, what changes in the service would make it easier for you or your family/carers to make your preferences known?
8. From your perspective, what changes in the service would make it easier for you or your family/carers to raise questions and concerns that you have about your care?
9. Do you have any other thoughts or comments you wish to add?

Thank the participant, close off the interview and provide information regarding the next steps in the process – when and how they can view the results and ask if they wish to be contacted to receive information regarding participation in the next stage of the study.
