# Supplementary material for: Improving quality and safety of cancer care for people from ethnic minority backgrounds: what do consumers want?
Source: Support Care Cancer. 2025 Jun 27;33(7):635. doi: 10.1007/s00520-025-09665-6 (PMC12204909; doi:10.1007/s00520-025-09665-6)
Supplement: Supplementary file 2 — Supplementary file2 (DOCX 17 KB) [file 520_2025_9665_MOESM2_ESM.docx]

*Supplementary File 1 : Participant Characteristics*

| ***Location*** | ***Gender*** | ***Ethnicity/Language Group identified*** | ***Language Support*** | ***Patient or family member*** | ***Interview Length (minutes)*** |
| --- | --- | --- | --- | --- | --- |
| NSW | F | Vietnamese | No | Patient | 30 |
| NSW | F | Arabic speaking | No | Patient | 41 |
| NSW | F | Arabic speaking | Yes – interpreter | Patient | 29 |
| NSW | F | Arabic Speaking | Yes- interpreter | Patient | 37 |
| NSW | F | Arabic speaking, Iraq | Yes- interpreter | Patient | 48 |
| NSW | M | Arabic speaking, Iraq | Yes- bilingual field worker | Patient | 39 |
| NSW | M | Arabic speaking | Yes- interpreter | Family member | 41 |
| NSW | F | Ukrainian | No | Family member | 46 |
| NSW | M | Chinese, Mandarin | Yes- bilingual worker | Patient | 76 |
| NSW | M | Chinese, Mandarin | Yes- bilingual worker | Patient | 65 |
| NSW | F | Arabic | No | Family member | NOTES ONLY – no recording |
| Vic | F | India | No | Patient | 24 |
| Vic | F | Arabic Speaking, Iraq | Yes | Patient | 33 |
| Vic | M | Iraq | No | Family member | 31 |
| Vic | F | Filipino | No | Family member | 42 |
| Vic | F | China | No | Patient | 28 |
| Vic | F | China | Yes- interpreter | Patient | 38 |
| Vic | F | China | No | Family member | 35 |
| Vic | F | China | Yes- interpreter | Patient | 32 |
