# Supplementary material for: Improving quality and safety of cancer care for people from ethnic minority backgrounds: what do consumers want?
Source: Support Care Cancer. 2025 Jun 27;33(7):635. doi: 10.1007/s00520-025-09665-6 (PMC12204909; doi:10.1007/s00520-025-09665-6)
Supplement: Supplementary file 3 — Supplementary file3 (DOCX 16 KB) [file 520_2025_9665_MOESM3_ESM.docx]

| 1. Considering language and culture in cancer care | - 1. *Interpretation of a cancer diagnosis*   2. *Support from people with a shared cultural background is valuable*   3. *Isolation can be a feature of immigration that impacts access to support*   4. *Raising issues or asking questions can be challenging* |
| --- | --- |
| 1. Facilitating opportunities to establish communication | - 1. *Negotiating appropriate communication options*   2. *Enabling use of interpreters*   3. *Translated information to support understanding and informed choice*   4. *Health service contact points between appointments* |
| 1. Improving the ability of health systems to accommodate difference | - 1. *Intractability of administrative processes*   2. *Information about services*   3. *Culturally appropriate supports* |

*Supplementary file 3: Themes and subthemes identified from the interview data*
